# A Feasibility Study of Co-Established Patient-Derived Subcutaneous Xenograft and Organotypic Slice Cultures in Hormone-Naive Primary Prostate Cancer Preclinical Modeling: A Single-Institution Experience

**DOI:** 10.3390/life15111719

**Published:** 2025-11-06

**Authors:** Valeria Pecci, Melissa Borsa, Aurora Aiello, Sara De Martino, Luca Cis, Francesco Pierconti, Domenico Varacalli, Martina Bracco, Cristian Ripoli, Francesco Pinto, Dante Rotili, Claudio Grassi, Carlo Gaetano, Alfredo Pontecorvi, Antonella Farsetti, Simona Nanni

**Affiliations:** 1 Department of Translational Medicine and Surgery, Università Cattolica Del Sacro Cuore, 00168 Rome, Italy; valeria.pecci@unicatt.it (V.P.); melissa.borsa@unicatt.it (M.B.); drvaracalli@icloud.com (D.V.); martibracco@icloud.com (M.B.); francesco.pinto@unicatt.it (F.P.); alfredo.pontecorvi@unicatt.it (A.P.); 2 National Research Council (CNR)-IASI, 00185 Rome, Italy; aurora.aiello@cnr.it (A.A.); sarademartino@cnr.it (S.D.M.); lucacis@cnr.it (L.C.); antonella.farsetti@cnr.it (A.F.); 3 Fondazione “Policlinico Universitario A. Gemelli IRCCS”, 00168 Rome, Italy; francesco.pierconti@unicatt.it (F.P.); cristian.ripoli@unicatt.it (C.R.); claudio.grassi@unicatt.it (C.G.); 4 Department of Woman, Child and Public Health, Università Cattolica Del Sacro Cuore, 00168 Rome, Italy; 5 Department of Neuroscience, Università Cattolica Del Sacro Cuore, 00168 Rome, Italy; 6 Department of Science, “Roma Tre” University, 00146 Rome, Italy; dante.rotili@uniroma3.it; 7 Biostructures and Biosystems National Institute (INBB), 00165 Rome, Italy; 8 Laboratory of Epigenetics, Istituti Clinici Scientifici Maugeri IRCCS, 27100 Pavia, Italy; carlo.gaetano@icsmaugeri.it; 9 Departmental Faculty of Medicine, UniCamillus—Saint Camillus International University of Health and Medical Sciences, 00131 Rome, Italy

**Keywords:** preclinical tumor model, surgical tissue explant, prostate cancer, translational research

## Abstract

Background: Preclinical models that preserve the tumor microenvironment are critically needed in prostate cancer (PCa) research. Patient-derived xenografts (PDXs) and patient-derived Organotypic Slice Cultures (PD-OSCs) have emerged as promising in vivo and ex vivo platforms to address this gap and better mimic human tumor biology. Methods: Subcutaneous PDX models and PD-OSCs were established in parallel from fresh, primary hormone-naïve PCa patient tissues. PDX models were generated by engrafting tumor fragments into immunodeficient mice, while PD-OSCs were maintained as short-term ex vivo cultures for functional analysis. Results: A cohort of 64 PDXs and 45 PD-OSCs was generated. While first-generation PDX engraftment was successful, subsequent passaging and model expansion were extremely poor. In contrast, PD-OSCs were reliably established, maintained tissue viability, and proved to be a robust platform for functional testing, including gene expression analysis and drug sensitivity screening. Conclusions: Our findings establish both first-generation PDXs and PD-OSCs as valuable “avatar” models for translational research. However, PD-OSCs represent a more efficient and rapid platform for studying primary hormone-naïve PCa biology and evaluating treatment responses, holding significant promise as a predictive tool to guide personalized medicine.

## 1. Introduction

Prostate cancer (PCa) continues to be a significant global health challenge, being one of the most frequently diagnosed malignancies and a leading cause of cancer-related mortality among men worldwide [1]. Despite success in the early stage with standard treatments (surgery, radiotherapy), about 30% of cases relapse to advanced and metastatic stages with no curative effect [2,3]. In the field of prostate cancer, there is the need for valuable preclinical models that can recapitulate disease progression, therapeutic resistance, and tumor heterogeneity.

Animal models represent useful in vivo experimental models for studying PCa biology and testing therapeutic approaches for translating preclinical results into clinical applications [4]. A lot of effort was dedicated to develop in vivo models that recapitulate human PCa carcinogenesis [5,6].

Among others, patient-derived xenografts (PDXs) are regarded as more effective in recapitulating the histopathological and molecular diversity of human cancer compared to other preclinical models [7,8].

PDXs are established by implanting tumor material into immunodeficient mice, generally by subcutaneous inoculation, with the major advantage of the integrity of the original tumoral tissue, which remains viable for weeks or months, thus providing valuable information on biology, microenvironment, biomarkers, and treatment sensitivity. In addition, PDXs may be serially transplanted after harvesting the first graft and re-implanting tissue to grow in subsequent host mice [9]. However, the PDX model for prostate cancer remains inadequate mainly because it is derived from metastatic tissues [10] or advanced disease, such as the largest collection of PDXs by the Movember GAP1 PDX international consortium that included PDXs from castration-resistant primary and metastatic prostate adenocarcinomas, as well as tumors with neuroendocrine differentiation [11].

In the prostate cancer research field, there is a significant need to establish a preclinical model recapitulating the heterogeneity and complex microenvironment of human PCa. The simple engraftment for PDX is the subcutaneous implant, with lower costs and easy execution. However, serially transplanted PDXs from primary hormone naive prostate cancer in subcutaneous models are rare and reported unsatisfactory efficiency [12,13,14,15,16].

Patient-derived Organotypic Slice Cultures (PD-OSCs) of fresh PCa tissues complement the in vivo PDX systems, providing an ex vivo platform that preserves the native tumor architecture and the tumor microenvironment (TME). PD-OSCs represent a relevant three-dimensional model recapitulating the specific characteristics of the original tissue with preservation of cancer, immune and stromal cell compartments and the overall cellular interactions [17,18]. PD-OSCs are obtained from fresh tissues explanted from prostate tumors during surgery and represent a short-term ex vivo culture capturing prostate cancer’s complexity and heterogeneity across patients [19,20,21,22,23], allowing researchers to gain insight into the different tumors coming from a heterogeneous population and providing a high-throughput and cost-effective model that retains the native tissue architecture [24]. The possibility to use PD-OSCs to profile key biomarkers or to correlate drug response to tumor pathology highlights their potential for use in precision oncology.

**Figure 1:**
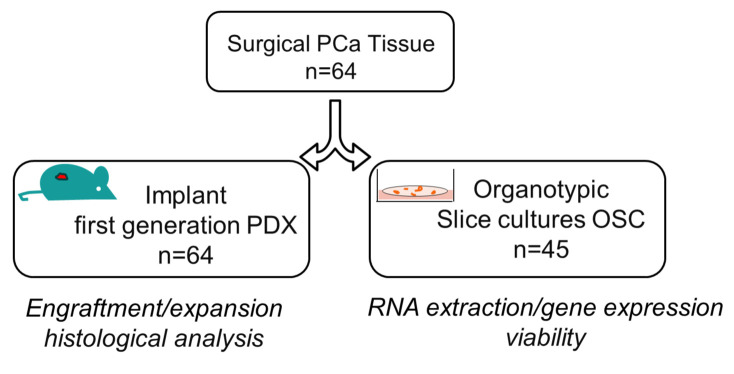
Overview of co-establishment of prostate cancer patient-derived xenografts (PDXs) and Organotypic Slice Cultures (OSCs). The surgically resected tumor specimen of hormone-naïve prostate cancer was divided into two pieces: one fragment was subcutaneously implanted in immunodeficient mice for the first generation of patient-derived xenograft (PDX, n = 64) while the other was sliced into thick sections for the generation of Organotypic Slice Cultures (OSC, n = 45). Characterization and analyses of both preclinical models are indicated.

Here, we co-established and characterized both PDX and PD-OSC models from hormone-naïve PCa patients, evaluating their feasibility and potential for translational research (Figure 1). Specifically, we generated 64 PDX engrafted tumor cohort from primary hormone naïve prostate cancer by analyzing tumor expansion after the first subcutaneous implant PDX (F1) and the serially transplanted engraftment (F2/F3), and comparing the results in two immunocompromised mouse strains, NOD/SCID and NSG, and two types of tissues, freshly explanted or cryopreserved. Overall, we observed a good engraftment in the first generation when using fresh tissues in NSG mice while subsequent re-implants were poor.

In addition, we established a parallel cohort of 45 PD-OSCs, derived from a sub-group of the 64 PDXs, evaluating the short-term vitality, the basal and the androgen-induced gene expression. The PD-OSC cohort was also useful to evaluate the drug response, ranging from a minimum of one to a maximum of four drugs evaluated. Overall, we developed an efficient experimental method to represent primary hormone-naïve prostate cancer providing evidence for its clinical predictivity.

## 2. Methods

**Human prostate cancer specimens**. PCa patients (n = 64) were enrolled at the Urology Clinic of IRCCS Fondazione Policlinico Gemelli—Università Cattolica of Rome, Italy to undergo prostatectomy between January 2020 to October July 2023 with the following inclusion criteria: (i) clinically localized PCa at diagnosis and (ii) absence of hormone treatment/radiotherapy before surgery. Freshly explanted tumor from the surgery was macro dissected by an expert pathologist and cut into 15 to 20 mm^3^ pieces. Tumor specimens were checked in sections adjacent to the engrafted samples for morphology, tissue architecture, and amount of tumor (≥75%). Fresh explant tissue was collected in medium and divided into two sections, one to generate OSCs (see paragraph below) and one transferred immediately to the animal laboratory for implanting in immunodeficient mice or frozen in FBS + 10% DMSO for further implanting, as described in the Results section.

**Tumor implantation and PDX mouse model.** NOD/SCID (RRID: IMSR_JAX:001303) and NSG (RRID: IMSR_JAX:005557) mice were obtained from Charles River Laboratories and housed 3–4 per cage in a room with controlled temperature, constant humidity, and a 12 h light/dark cycle with free access to food and water. Tumor fragments, placed in medium and coated in Matrigel, were subcutaneously implanted in male 6–8 week old NOD/SCID or NSG mice (see Results section) by a small incision and subcutaneous pocket made in one side of the lower back to obtain the first patient-derived xenograft PDX generation (F1) [25]. Tumor growth was measured with digital calipers, and tumor volumes were calculated from the formula V = (w^2^ × l)/2; w = width and l = length. After 4–6 months (or at 1000 mm^3^ tumor volume [26,27]), subcutaneous tumors were removed and divided into 2/4 pieces: one collected for histology and the remaining for further transplant to obtain the second PDX generation of mice (F2). Same procedures were conducted to obtain the third PDX generation (F3). Engraftments and original prostatic surgical specimens were compared using histology and immunohistochemistry.

**Histological analysis**. For histological analyses, tissues were fixed in 10% formalin (Thermo Fisher Scientific, Waltham, MA, USA). Unstained tissue sections (4 μm-thick) were cut from formalin-fixed, paraffin-embedded blocks and mounted on a positively charged glass slide. Hematoxylin and eosin staining was performed according to standard procedures. Tissue specimens were examined using Olympus cellSens platform, SC30 microscope camera (Olympus, Tokyo, Japan) with cellSens Entry software version 2.3 for digital image acquisition.

**Patient-derived Organotypic Slice Cultures (PD-OSCs).** Fresh explants of PCa tissues (n = 45) were used to generate OSCs as we previously described in [19,20,21,22,23]. Briefly, fresh tissues were immediately sliced into thick sections (350 μm) using McILWAIN TISSUE CHOPPER (Campden Instruments, Loughborough, England), then cultured for 72 h at a liquid–air interface using semi-porous tissue culture inserts (PICM03050, Millipore Darmstadt, Germany) placed in a six-well plate using 5 slices/insert. Slices were incubated in a 37 °C humidified incubator with 5% CO_2_. Medium was changed daily. Slices were treated with DHT (10^−7^ M) for 72 h, vehicle (ethanol) was used as control.

**Cell cultures.** PC-3luc (RRID:CVCL_J265) from the American Type Culture Collection in December 2024 were grown in FK-12 (Life technologies, Carlsbad, CA, USA) medium supplemented with 10% FBS according to instructions. PrEC (RRID:CVCL_0061) cells were obtained from Lonza in January 2023 and were grown in PrEGM (Lonza, Walkersville, MD, USA) medium supplemented with a bullet kit according to the instructions. Indirect (Hoechst 33342, Thermo Fisher Scientific) methods routinely screened all cell lines for mycoplasma contamination. Treatment: etoposide (Merk, Darmstadt, Germany) was dissolved in DMSO and used 10 microMolar for 72 h

**Cell death assay.** Cell death was measured using the cell death detection ELISA PLUS kit (Roche, Basel, Switzerland) according to the manufacturer’s instructions. Briefly, extracellular-medium (5–20 µL) was incubated with anti-histone-biotin antibody and anti-DNA-peroxidase antibody in a streptavidin-coated 96-well plate on an orbital shaker (80 rpm) in the dark at room temperature for 2 h. Absorbance at 405 and 490 nm was assessed at VICTOR X4 (Perkin Elmer, Waltham, MA, USA) to quantify histone-associated DNA fragments released during apoptosis, necrosis, or other forms of cell death.

**RNA extraction, cDNA preparation, and real-time PCR.** RNA was extracted with 1 mL Trizol (Thermofisher) using tissue homogenizer iRUPT (Neuation Technologies, Ahmedabad, India) according to the manufacturer’s instructions. The RNA pellet was resuspended in 10 µL of RNase-free water and incubated at 65 °C for 10 min. RNA concentration was determined using a NanoDrop spectrophotometer with evaluation of A260:A280 ratio for quality assessment: samples with a A260:A280 ratio of 1.8–2.0 were included in further analysis. cDNA was prepared using 2 micrograms of RNA and quantitative real-time PCR was performed as in [23] on QuantStudio 7 Pro Real-Time PCR System (Applied Biosystems, Foster City, CA, USA) using SYBR Green quantification. The relative amount of each gene was measured as 2 − ΔCt. β-Actin, P0 or GAPDH served as endogenous control. Primers to P0 and GAPDH were as in [21], PSA and β-Actin as in [19], and as follows: AR 5′-TCACCCCCCAGGAATTCC-3′ and 5′-ATGATACGATCGAGTTCCTTGATG-3′, GLUT1 5′-CGGGCCAAGAGTGTGCTAA-3′ and 5′-TCCTTCATCTCCTGCAGGTCA-3′, PS2 5′-CATCGACGTCCCTCCAGAAGAG-3′ and 5′-CTCTGGGACTAATCACCGTGCTG-3′.

**Statistical analysis.** Data were expressed as mean ± SEM or as fold change as indicated in the figure legends. The differences among ≥ 3 groups were analyzed with a Kruskal–Wallis test, and post hoc comparison was performed using the Mann–Whitney U test (α  =  0.05). The differences among two groups were analyzed with the Mann–Whitney U test. The Pearson correlation was used to measure the linear correlation between two data sets. The efficiency of both PDX engraftment/expansion and PD-OSC gene expression by qPCR was evaluated as categorical variables (successful vs. unsuccessful) and comparisons were assessed using the Chi-square test and the contingency coefficient to evaluate the strength of association between categorical outcomes. Logistic regression analyses were conducted to examine the relationships between clinical variables (age, serum PSA, ISUP grade, recurrence) and the probability of successful PDX engraftment/expansion and OSC gene expression. Univariate analyses were initially performed, followed by multivariate logistic regression to identify independent predictors for variables that were statistically significant at the univariate analysis. Statistical analysis was performed using GraphPad Prism 8.0.2 statistical software or R software (version 4.5.0). *p*-values < 0.05 were considered significant.

## 3. Results

### 3.1. Development of Patient-Derived Xenografts (PDXs)

We aimed to develop a patient-derived xenograft (PDX) tumor mouse model that allows the propagation and expansion of tumors, offering relevant predictive insights when evaluating the efficacy of novel cancer therapies. According to the literature, we expected a take rate around 25% of the prostate cancer PDX-engrafted cohort [10,28]

Tissue samples were collected from sixty-four prostate cancer patients who underwent radical prostatectomy, from January 2020 to October 2023, for primary non-metastatic prostate cancer; furthermore, all patients were hormone-naïve [Table 1 and Table S1, Age (years): mean 69.2, range 49–80; pathological GS ≤ 7 (3 + 4) n = 45, GS ≥ 7 (4 + 3) n = 19]. The disease’s clinical progression was defined by the presence of biochemical, local, or metastatic recurrence (n = 23 out of 64, follow-up range 15 months–5 years).

PCa-derived tissues were implanted subcutaneously into a total of 7 NOD/SCIDs and 64 NSG mice to generate PDX tumor-engrafted cohorts. Specifically, freshly explanted tumors were subcutaneously implanted in male 6–8 week old mice as described in the Methods section. Initially, fresh tissues from the 7 patients, enrolled from January to July 2020, were implanted in NOD/SCID mice (F1 generation, n = 7) but no expansion of tumors was observed in the expected time (about 4/6 months). Next, we refined methods of performing implants on NSG mice using both frozen tissues, collected from January 2020 to February 2021, or fresh tissues, collected from February 2021 to October 2023 (total F1 generation n = 64, 20 from frozen, 44 from fresh tissues, Table 2). Slow tumor expansion was observed only in 2/20 NSG mice who received frozen tissues. As the best result, tumor expansion and palpable engraftment were observed in 16/44 NSG mice [take rate 36%, according to the expected PCa take rate (25–30%)] received from fresh tissues within 3–4 months post-implant. Six months after the implant, 7 PDX-F1s were explanted at 1000 mm^3^ to produce the F2 generation (n = 3 mice/each) and only 3 PDX-F2s were explanted to obtain the F3 generation (n = 3 mice/each).

Of note, tumor engraftment, and expansion at the F1 generation appears not to be associated with patient recurrence: only six out of sixteen palpable engraftments were from PCa patients who experienced recurrence. Similarly, the tumor take rate was not correlated with the Gleason score, pathological stage, patient age, or PSA level using logistic regression.

**Figure 2:**
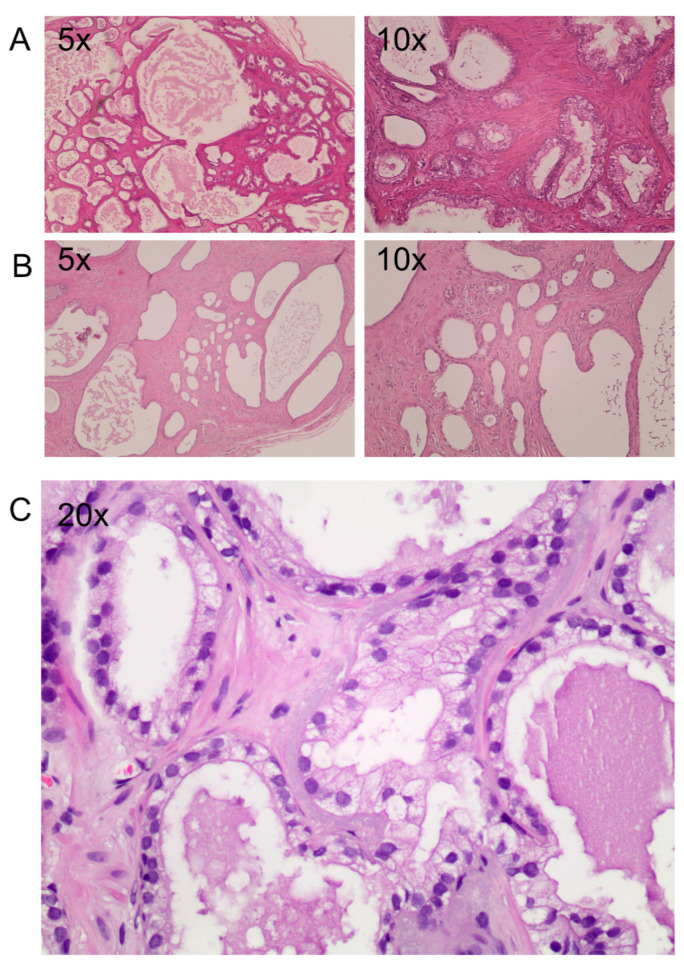
**Histological staining of two engraftments.** (**A**–**C**) Hematoxylin and eosin staining of two PDXs 6 months after implants at different magnification (5×, 10× and 20×). (**A**) An implant with a prostate cancer area maintaining the original Gleason score. (**B**) Atrophic tissue with a failing cytoplasmic area. (**C**) Atypical gland among normal glands with a mono-stratified epithelial layer, enlargement of the nuclei, presence of central nucleolus and absence of basal cells.

Interestingly, the tissue architecture, evaluated by H&E staining, confirmed that the F1 generation maintains prostate cancer pathological and morphological features with a good preservation of epithelium–stroma compartments (Figure 2).

### 3.2. Establishment of Patient-Derived Organotypic Slice Cultures (PD-OSCs)

To define a reliable preclinical model of hormone-naïve prostate cancer, we generated a cohort of patient-derived Organotypic Slice Cultures (PD-OSCs) starting from the same patients described above (Table 1 and Table S1). From a subset of PCa patients (n = 45) depending on the available tissue, we were able to establish the ex vivo preclinical model PD-OSCs that preserve the human tumor architecture and microenvironment. Figure S1 shows a representative OSC derived from fresh PCa tissue depicting four different inserts with 5 slices/inserts enabling them to test four different experimental conditions (e.g. drug, compounds, hormones, etc.).

**Figure 3:**
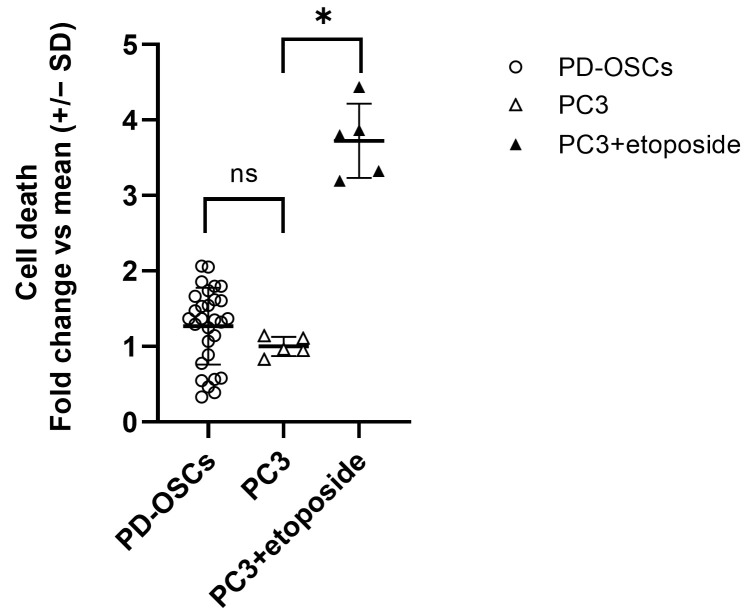
**Vitality of PD-OSCs**. Cell death was assessed by cell death detection ELISA on an extracellular medium collected after 72 h culture of PD-OSCs (n  =  29); PC3 and etoposide-treated PC3 cells served as control. Data expressed as fold change vs. mean PC3 are plotted as mean +/− SEM, individual values are indicated as dots (PD-OSCs) . * *p* < 0.05, ns = not significant.

Viability was evaluated by quantification of degraded DNA fragments released by apoptotic or necrotic cells. As shown in Figure 3, the viability of PD-OSCs after 72 h in culture is comparable to that observed in standard cell line culture which occurred in PC3 cells at 80% confluence. Treatment of PC-3 cells with the topoisomerase II enzyme inhibitor etoposide (10 µM for 72 h) was used as positive control for cell death detection.

**Figure 4:**
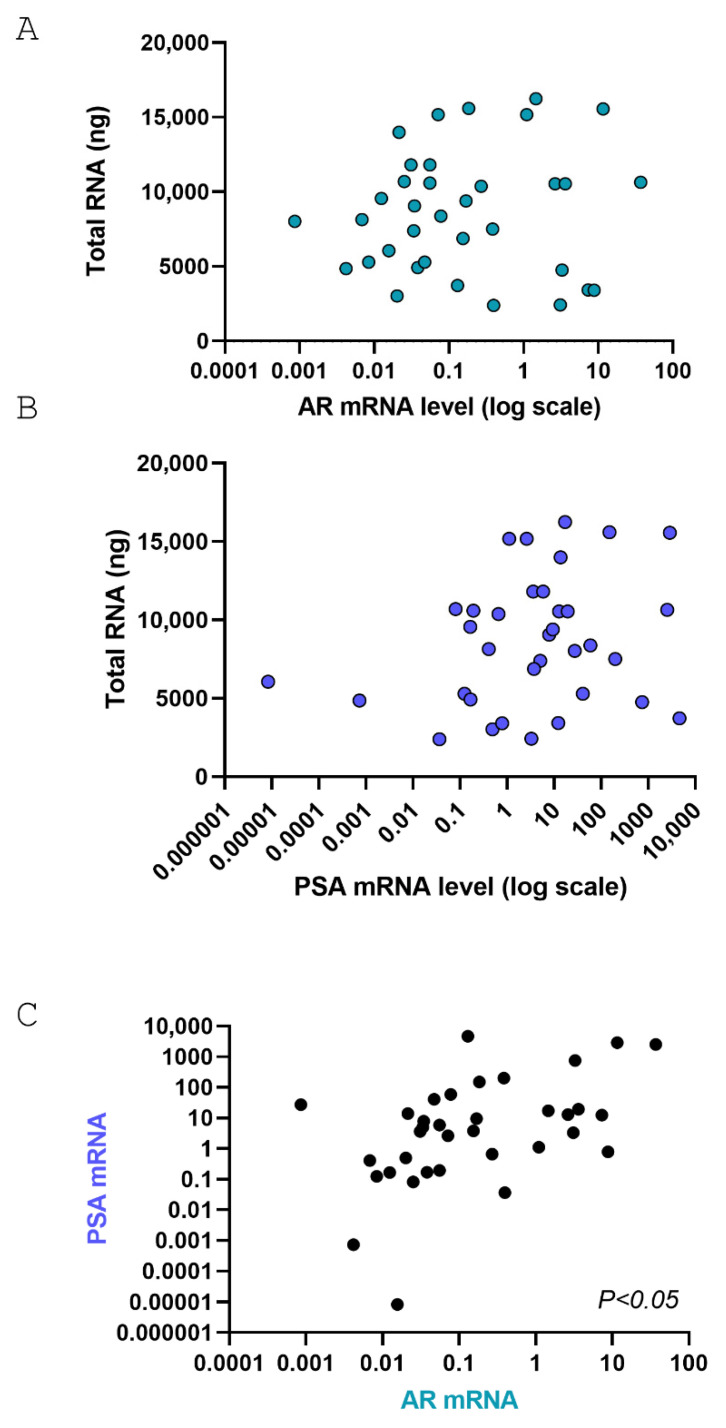
Pearson correlation analysis between AR and PSA in PD-OSCs. (**A**,**B**) Pearson correlation between total RNA extracted and AR mRNA levels (**A**, *p* = not significant) or PSA mRNA levels (**B**, *p* = not significant); (**C**) Pearson correlation between AR and PSA mRNA levels after 72 h cultures in PD-OSCs (n = 35; *p* < 0.05).

For gene expression analysis, total RNA was extracted with Trizol obtaining a total high-quality RNA recovery ranging from 2 to 16 micrograms in 35/45 samples. Two micrograms of RNA were retrotranscribed and cDNA was used to quantify androgen receptor (AR) and PSA mRNA levels by Real Time PCR. Levels of AR or PSA are not associated with total RNA recovery while, as expected, levels of PSA and AR showed a positive association (Figure 4).

**Figure 5:**
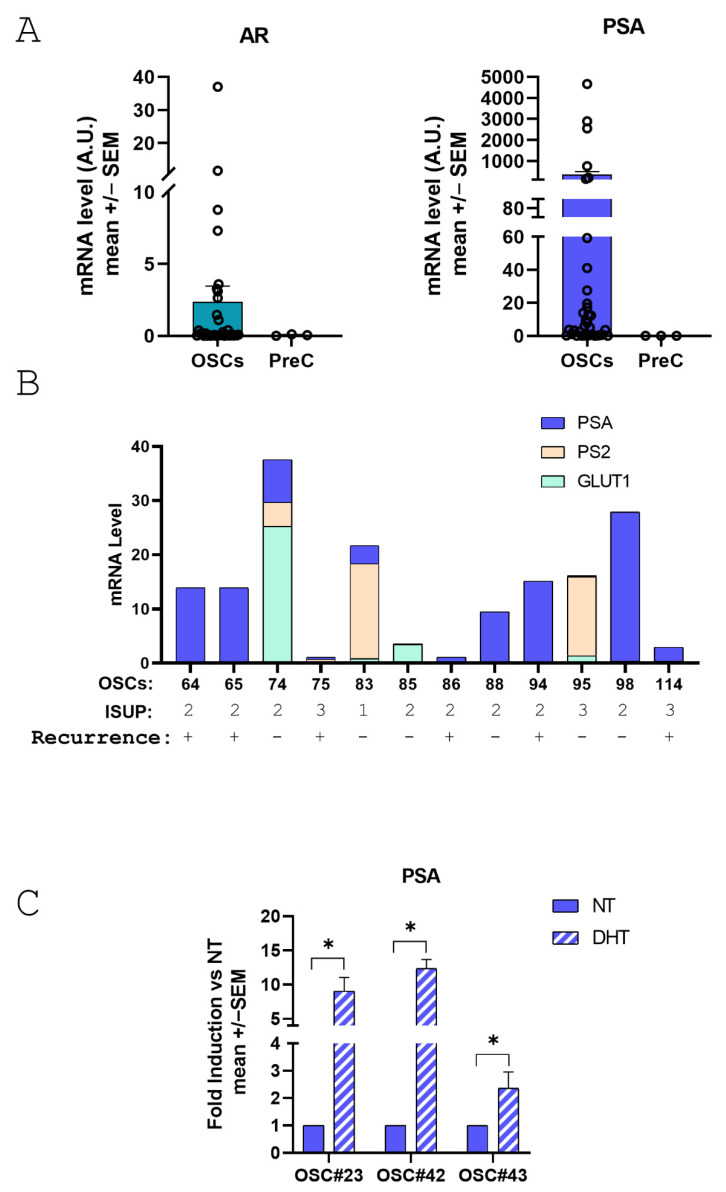
**Expression of androgen receptor(AR), prostate specific antigen (PSA) and GLUT-1 in PD-OSCs and response of PSA to androgen treatment**. (**A**) AR (left) and PSA (right) expression were quantified by qRT-PCR in PD-OSCs (OSC, n = 35) compared to PrEC cells as negative control. Data, expressed as mean +/− SEM, represent normalized values in OSC and PrEC of AR and PSA, respectively. A.U., arbitrary unit. (**B**) mRNA levels of GLUT-1, PS2 and PSA in individual OSCs plotted as a stacked column chart. ISUP grade and recurrence status are indicated. (**C**) PSA mRNA level was assessed in three different OSCs after 24 h treatment with DHT (10^−7^ M) compared to control (NT). Data are plotted as fold induction vs. NT (mean +/− SEM). * *p* < 0.05 DHT vs. NT.

It is of note that levels of AR and PSA are nicely preserved in PD-OSCs after 3 days in culture compared to negative control PrEC cells derived from undifferentiated basal epithelial cells (Figure 5A). Interestingly, within a subset of OSCs with similar biological properties of the whole patient’s cohort in terms of ISUP and Recurrence, we observed that different PD-OSCs express different levels of several TME biomarkers such as the glucose transporter protein type 1 (GLUT-1) as the hypoxia target gene, PS2 as the estrogen target gene and PSA as the androgen target gene (Figure 5B). These findings highlighting the difference among the original PCa tissues/patients reflect the well known intertumoral heterogeneity of primary prostate cancer. Lastly, to assess whether the androgen responsiveness of the original tumor tissue was preserved in OSCs, three different PD-OSCs were treated with DHT. PSA mRNA induction was used as proof-of-principle. The PD-OSC system nicely preserves response to the androgen hormone as assessed by PSA induction after a 24 h treatment with DHT (Figure 5C).

In our experimental context, two inserts with five slices/each were obtained from 27% of PCa tissues, 3 inserts from 29% and 4 inserts from 33% of cases allowing to assess from 1 to 3 different drugs compared to control DMSO (Figure S1 and Table 3). In about 10% of cases, it was possible to establish five inserts from the same patient to test 4 different drugs at the same time. Of note, the number of inserts appears not to be correlated with patient recurrence.

One major advantage of utilizing the PD-OSC system in translational research is the possibility to manipulate PCa tissues with several methodologies including transfection, interference and treatment with tumor micro-environment stimuli and/or small molecules (epidrugs). Table 4 summarizes our past experience in handling fresh PCa tissues as a patient-derived Organotypic Slice Culture (PD-OSC) system while current experience resides in the co-establishment of PDX and PD-OSC preclinical models.

These results highlight the potentiality of this experimental model as a platform to test novel anti-cancer therapies revealing differences between PCa and patients’ responses, and deepen intertumoral heterogeneity across different tumors from different individuals, even with similar histopathological characteristics.

**Figure 6:**
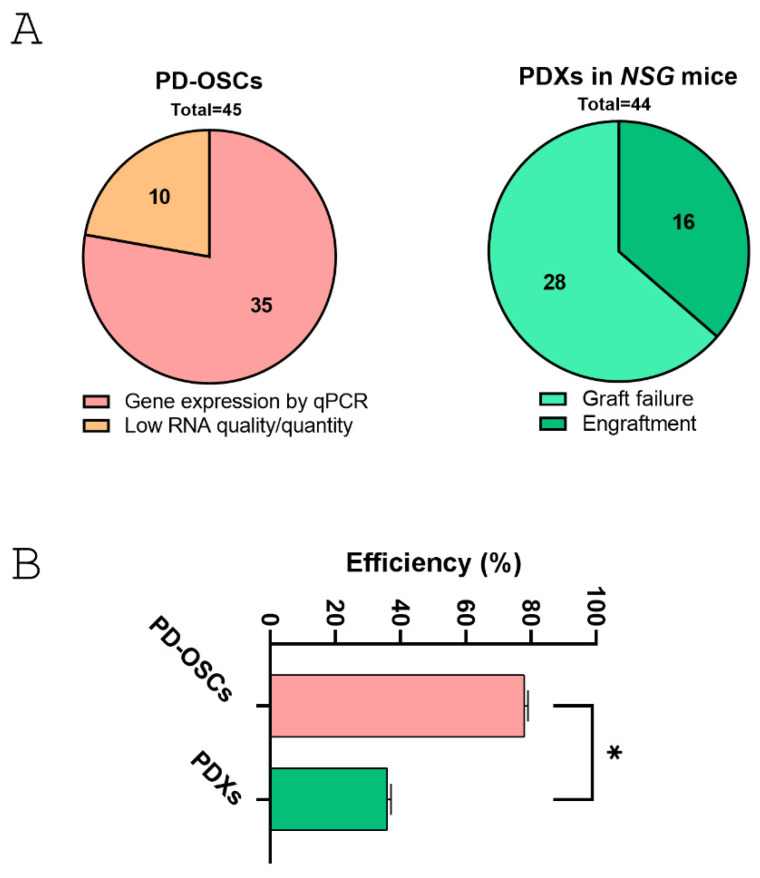
**Comparative analysis between co-established PDX and PD-OSC preclinical models in hormone-naïve prostate cancer.** (**A**) The pie charts show the successful outcome in PD-OSCs (gene expression by qPCR vs. low RNA quantity/quality, left) and in PDXs derived from fresh tissue implants in NSG mice (engraftment vs. graft failure, right). (**B**) Efficiency rate (expressed as percentage) for the two systems. Statistical significance was assessed using contingency analysis. * *p* < 0.05.

Overall, the co-establishment of hormone-naive primary prostate cancer-derived PDX and PD-OSC models provides a realistic tumor microsystem for translational research. Both systems maintain tissue architecture and 3D structure. However, PD-OSCs offer a more applicable and rapid platform than PDX. In terms of efficiency, a direct comparison between successful engraftment/expansion in PDX models and satisfactory gene expression by qPCR in PD-OSCs, revealed that the PD-OSC system is significantly more effective than PDX (Figure 6). The overall efficiency was 78% for RNA extraction and gene expression in PD-OSCs and 36% for PDX engraftment in NSG mice in the first generation. The contingency analysis revealed a highly significant difference between the two systems (χ^2^ = 15.59, DF = 1, *p* < 0.001).

The achievement of a successful PD-OSC system was independent from clinical and histopathological parameters (age, serum PSA, ISUP) or disease progression (recurrence) evaluated using logistic regression, emphasizing the relevance of such a system for hormone-naïve prostate cancer modeling.

## 4. Discussion

Prostate cancer is characterized by heterogeneous cellular populations and a highly dynamic microenvironment which together drive tumor progression and resistance to therapy. Developing preclinical models that accurately recapitulate heterogeneity and complexity in human PCa is a critical need, particularly for models that preserve the native tumor microenvironment in hormone-naïve PCa. In this direction, patient-derived xenograft and Organotypic Slice Cultures have gained attention as they more closely recapitulate the three-dimensional (3D) tissue architecture and tumor microenvironment compared to conventional 2D cell culture systems. These preclinical models allow us to preserve the overall cell–cell and cell–matrix interactions in the original tissue, which are crucial for understanding in vivo cancer cells’ behavior and for exploring drug efficacy and resistance mechanisms in a more physiological context compared to traditional models.

Here we described the successful co-establishment of both patient-derived xenografts (PDX, n = 64) and Organotypic Slice Cultures (PD-OSCs, n = 45) from the same surgical explant of fresh hormone-naïve prostate cancer tissue. Of interest, our patient cohort is representative of the overall prostate cancer population where most patients (57/64) belong to the relatively homogeneous clinical and pathological ISUP 2 (Gleason Score 3 + 4) and ISUP 3 (Gleason Score 4 + 3) classes, and about 30% of patients (23/64) presented disease recurrence after surgery.

The main results regarding our PDX model of hormone-naïve prostate cancer reside in the successful engraftment and growth of the first generation of implants in mice. On the contrary, the second and third PDX generations exhibited insufficient expansion. Despite several studies on PDXs as efficient PCa preclinical models [29], our data report a limited ability of the PDX model for prostate cancer therapeutic testing due to the impossibility to obtain the necessary number of animals to perform an adequate drug testing response. These results are in line with a previous report by Valta et al. 2020 that also showed a decline of tumor engraftment at the second and third PDX generation [15]. Although an improvement in PDX engraftment has been observed in prostate cancer using more severely immunodeficient strains [11], we and others do not find benefits from using them [13]. The use of first-generation PDXs currently appears to be the only possibility in the prostate cancer field, where the challenge to serially transplant PDXs remains unsolved [9].

On the contrary, we developed a reliable PCa preclinical model, useful to test novel targeted therapy, consisting of a cohort of patient-derived Organotypic Slice Cultures established from fresh explants of hormone-naïve prostate cancer, which preserved tissue viability and responsiveness to treatments. As main results from this second model, we demonstrated that five slices/inserts yield sufficient material for gene expression analysis and evaluation of viability/cell death. Of note, the use of five different slices/inserts relieves and attenuates the well-known structural variation within the tumor, specifically in the case of PCa. In addition, the system allows to generate at least two inserts from each PCa specimen, with a maximum of five inserts, enabling the testing of different drugs [22,23]. These data are in line with previous results reporting OSCs as a relevant and functional model to study the intrinsic properties of PCa and as a useful tool for the identification of targeted cancer treatment [17,18,30]. Furthermore, such a preclinical model preserves the response to tumor microenvironment stimuli like hypoxia [21,30] and to steroid hormones or antagonists [19,21,31,32] as well as to DNA damage agents like chemotherapeutics or ionizing radiation [33]. While offering rapid the evaluation of drug sensitivity in a preserved tumor microenvironment, the short-term culture of PD-OSC limits the assessment of long-term responses [34].

Overall, our data demonstrated that a cohort of PD-OSCs represents a valid tool to mimic the response of prostate cancer to treatments and to be used as a predictive model for clinical outcomes highlighting the translational value of this model.

This experimental approach opens the doors to the development of a platform in which PD-OSCs derived from individual patients can be used to evaluate the sensitivity of tumors to different therapeutic agents with the potential to drive treatment decisions and improve patient outcomes. The response of a single OSC is indeed associated with the original tissue and therefore with the individual characteristics of PCa patients—a sort of avatar to predict the therapeutic responses in vivo. The possibility of observing different responses from different PD-OSCs, in turn PCa patients, provides the basis of a personalized model of primary prostate cancer treatment.

However, some limitations exist in using PD-OSCs in preclinical translation prostate cancer research. For instance, the maintenance of slice cultures requires specialized techniques to preserve tissue integrity and functionality, and the short-term nature of the cultures might limit their capability to fully capture long-term therapeutic responses. In addition, both subcutaneous PDXs and PD-OSCs are unsuitable in studying metastasis dissemination. Moreover, considering a certain degree of heterogeneity among sections, at least three slices under different experimental conditions should be used to account for intra-tumoral heterogeneity. However, at the same time, this characteristic of OSCs, when appropriately exploited, makes it a unique experimental model, not reproducible in organoids or murine models.

In conclusion, this study reports a direct comparison of PDX and PD-OSC preclinical models derived concurrently from the same patient cohort. The co-establishment of hormone-naive primary prostate cancer-derived models provides a realistic tumor microsystem for translational research in which PD-OSCs offer a more applicable, rapid, and predictive platform than PDXs. Our data address PD-OSCs as an effective experimental model capable of capturing the complexities of prostate cancer, a crucial step for advancing therapeutic strategies and improving patient outcomes covering the gap in preclinical models of prostate cancer. Although OSC methodology has existed for many years, such a platform has not been widely adopted. Our data highlights the possibility of using PD-OSCs in translational research providing evidence for its potential in cancer drug testing and biomarker discovery programs.

## Figures and Tables

**Table 1:** Prostate cancer cohort. Clinical and histopathological characteristics of PCa patient cohort.

	Patient Information Before Surgery		Clinical and Histopathological Features			Pre-Clinical Model		
Total Patient no.	Age (years) mean ± SD	Serum PSA (ng/mL) Mean ± SD	Pathological ISUP * score (%)	Pathological TMN (%)	Post-surgical recurrence (%)	PDX no.	PD-OSC no.	
64	69.2 ± 6.4	12.4 ± 7.3	ISUP 1 (9.4%) ISUP 2 (60.9%) ISUP 3 (26.6%) ISUP 5 (3.1%)	pT2c (64%) pT3a (23.4%) pT3b (12.5%)	23 (35.9%)	64	45	

* Prostate cancer grade group according to ISUP (International Society of Urological Pathology).

**Table 2:** Prostate cancer subcutaneous engraftment and tumor expansion in the PDX-F1 generation.

	Subcutaneous Implant				Tumor Expansion	
Number of Patients *	Mouse Strain	Fresh Tissue	Frozen Tissue	Number of Mice	Palpable Engraftment	Tumors Take Rate % (Palpable Engraftment/Number of Mice)
7 (PDX#1–7)	NOD/SCID	yes	-	7	0	0% (0/7)
20 (PDX#1–20)	NSG	-	yes	20	2	10% (2/20)
44 (PDX#21–64)	NSG	yes	-	44	16	36% (16/44)

* (*PDX#* as in Table S1).

**Table 3:** Multi-insert efficiency in PD-OSC establishment.

	Number of Inserts			
Number of Patients *	2 inserts (%)	3 inserts (%)	4 inserts (%)	5 inserts (%)
Total n = 45	12 (27%)	13 (29%)	15 (33%)	5 (11%)
No recurrence = 29	9 (31%)	8 (28%)	9 (31%)	3 (10%)
Recurrence n = 16	3 (19%)	5 (31%)	6 (38%)	2 (12%)

* PD-OSC as in Table 1.

**Table 4:** Manipulation and application of PD-OSCs.

Total OSCs	Methodology	Analysis	Reference (doi)
N = 9	Transfection/interference	- Gene expression/Real time PCR - Gene expression/Western blot	Aiello et al., 2016 [19] (doi: 10.1038/srep38414)
N = 8	Tumor microenvironment stimuli (hypoxia and estrogen)	- Gene expression/Real time PCR - Gene expression/Western blot	Bacci et al., 2019 [21] (doi: 10.3390/ijms20164012.)
N = 50	Transfection/interference	- Gene expression/Real time PCR - Gene expression/Western blot - Secretion lactate and citrate (extracellular medium)	Nanni et al., 2020 [20] (doi: 10.3390/cancers13010015)
N = 25	Small molecules (epidrugs)	- Gene expression/Real time PCR - Apoptosis (extracellular medium)	Pecci et al., 2024 [22] (doi: 10.1186/s12935-024-03231-6)
N = 28	Small molecules (epidrugs)	- Gene expression/Real time PCR - Chomatin -ImmunoPrecipitation (ChIP)	Pecci et al., 2025 [23] (doi: 10.3390/ncrna11030033)

## Data Availability

The original contributions presented in the study are included in the article; further inquiries can be directed to the corresponding author.

## Supplementary Materials

The following supporting information can be downloaded at: https://www.mdpi.com/article/10.3390/life15111719/s1, Figure S1. Representative Organotypic Slices Cultures (OSCs) derived from one patient (OSC#102) after 72 h treatment with DRUG#1, DRUG#2, DRUG#3 or vehicle as control (see also Table 4). OSCs were cultured on semi-porous tissue culture inserts (5 slice/insert); Table S1: Clinical and histopathological features of PCa patients used for PDXs and OSCs generation.
