# Supplementary material for: A Feasibility Study of Co-Established Patient-Derived Subcutaneous Xenograft and Organotypic Slice Cultures in Hormone-Naive Primary Prostate Cancer Preclinical Modeling: A Single-Institution Experience
Source: Life (Basel). 2025 Nov 6;15(11):1719. doi: 10.3390/life15111719 (PMC12653099; doi:10.3390/life15111719)
Supplement: Supplementary file 1 [file life-15-01719-s001.zip › figure S1_PDX paper_23ottobre2025_rev.pdf]

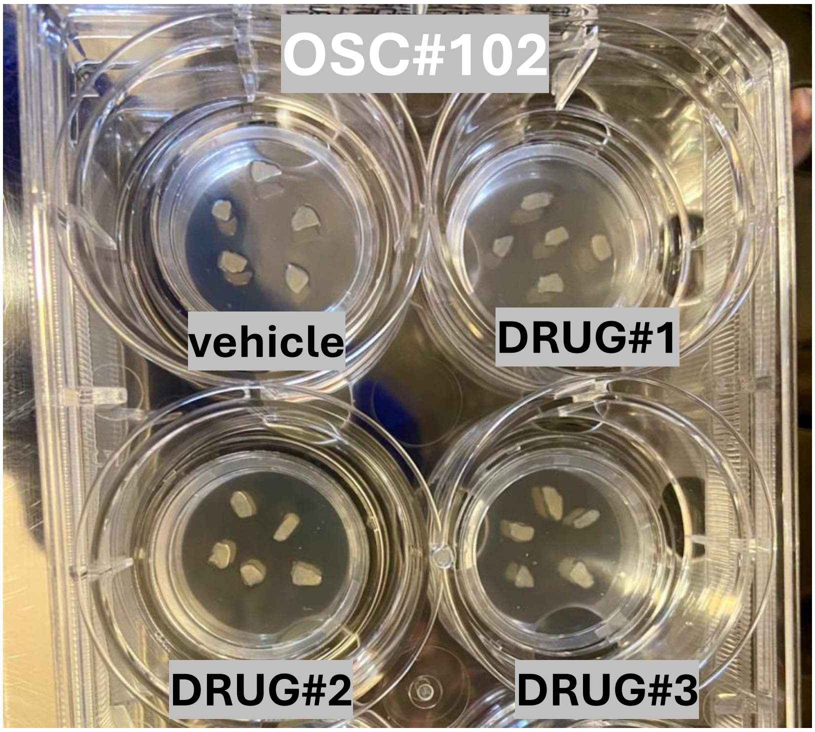

Figure S1. Representative Organotypic Slices Cultures (OSCs) derived from one patient (OSC#102) after 72 h treatment with DRUG#1, DRUG#2, DRUG#3 or vehicle as control (see also Table 4). OSCs were cultured on semi-porous tissue culture inserts (5 slice/insert).
