# Supplementary material for: A Feasibility Study of Co-Established Patient-Derived Subcutaneous Xenograft and Organotypic Slice Cultures in Hormone-Naive Primary Prostate Cancer Preclinical Modeling: A Single-Institution Experience
Source: Life (Basel). 2025 Nov 6;15(11):1719. doi: 10.3390/life15111719 (PMC12653099; doi:10.3390/life15111719)
Supplement: Supplementary file 1 [file life-15-01719-s001.zip › Table S1_R1_life_291025.pdf]

Table S1. Clinical and histopathological features of PCa patients used for PDXs and OSCs generation

	Patient information before surgery			*Clinical and histopathological features*				
Patient no.	Age (years)	PSA (ng/mL)	Gleason score biopsy	Pathological Gleason Score	Pathological ISUP*	Pathological TMN	Recurrence (months after surgery)	PD-OSC
PDX#1	72	6.2	6 (3+3)	7 (3+4)	2	pT3a pNx pMx	-	Yes
PDX#2	74	10	6 (3+3)	6 (3+3)	1	pT2c pNx pMx	-	
PDX#3	67	13.8	7 (3+4)	7 (3+4)	2	pT3a pN0 pMx	-	Yes
PDX#4	74	4.2	6 (3+3)	6 (3+3)	1	pT2c pNx pMx	-	
PDX#5	80	9.8	8 (4+4)	7 (4+3)	3	pT2c pN1 pMx	Yes (4)	
PDX#6	62	33	8 (4+4)	7 (4+3)	3	pT3a pNx pMx	Yes (3)	
PDX#7	69	7	7 (4+3)	7 (3+4)	2	pT2c pN0 pMx	-	
PDX#8	69	2.4	7 (3+4)	7 (3+4)	2	pT3a pNx pMx	Yes (6)	Yes
PDX#9	71	12	7 (3+4)	7 (3+4)	2	pT3a pNx pMx	-	
PDX#10	69	8.5	7 (3+4)	7 (3+4)	2	pT2c pNx pMx	-	
PDX#11	75	7.1	7 (3+4)	7 (3+4)	2	pT3a pN0 pMx	Yes (4)	Yes
PDX#12	60	6.2	7 (3+4)	7 (4+3)	3	pT3a pN0 pMx	Yes (2)	
PDX#13	67	20	6 (3+3)	7 (3+4)	2	pT2c pNx pMx	-	Yes
PDX#14	75	10.4	8 (4+4)	7 (3+4)	2	pT2c pN0 pMx	-	
PDX#15	80	15.2	8 (4+4)	9 (4+5)	5	pT3b pN0 pMx	Yes (3)	Yes
PDX#16	76	6.7	7 (4+3)	7 (3+4)	2	pT3a pN1 pMx	Yes (10)	Yes
PDX#17	73	8.2	6 (3+3)	7 (3+4)	2	pT2c pN0 pMx	-	Yes
PDX#18	75	8.4	7 (3+4)	7 (3+4)	2	pT2c pNx pMx	-	Yes
PDX#19	76	6.1	7 (3+4)	7 (3+4)	2	pT3b pNx pMx	-	Yes
PDX#20	72	14.7	9 (4+5)	7 (4+3)	3	pT3b pN0 pMx	Yes (2)	
PDX#21	65	7.6	7 (3+4)	7 (4+3)	3	pT2c pNx pMx	Yes (23)	Yes
PDX#22	70	2.4	6 (3+3)	6 (3+3)	1	pT2c pNx pMx	-	
PDX#23	66	4.6	7 (3+4)	7 (3+4)	2	pT2c pNx pMx	-	
PDX#24	70	8	6 (3+3)	7 (3+4)	2	pT2c pNx pMx	-	Yes
PDX#25	65	13	7 (3+4)	7 (3+4)	2	pT3a pN0 pMx	Yes (13)	Yes
PDX#26	75	7.6	7 (3+4)	7 (3+4)	2	pT2c pN0 pMx	Yes (2)	Yes
PDX#27	79	4.2	7 (3+4)	7 (3+4)	2	pT2c pNx pMx	-	
PDX#28	73	9.9	7 (4+3)	7 (4+3)	3	pT3b pN1 pMx	Yes (7)	Yes
PDX#29	69	9	8 (5+3)	7 (3+4)	2	pT2c pN0 pMx	-	Yes
PDX#30	69	5	7 (4+3)	7 (4+3)	3	pT3a pN0 pMx	-	Yes
PDX#31	68	5	7 (3+4)	7 (3+4)	2	pT3a pN0 pMx	-	Yes
PDX#32	64	6.7	6 (3+3)	7 (3+4)	2	pT2c pNx pMx	-	Yes
PDX#33	71	4.8	7 (4+3)	7 (3+4)	2	pT2c pN0 pMx	-	Yes
PDX#34	59	5	7 (3+4)	7 (3+4)	2	pT2c pNx pMx	-	Yes
PDX#35	70	13	8 (4+4)	9 (4+5)	5	pT3b pN0 pMx	Yes (3)	
PDX#36	57	5.4	7 (3+4)	7 (3+4)	2	pT2c pNx pMx	Yes (2)	Yes
PDX#37	69	3.9	7 (3+4)	7 (3+4)	2	pT2c pNx pMx	-	Yes
PDX#38	78	17	7 (3+4)	7 (4+3)	3	pT3a pNx pMx	Yes (8)	Yes
PDX#39	56	20	7 (4+3)	7 (4+3)	3	pT2c pN0 pMx	Yes (2)	
PDX#40	74	7	7 (4+3)	7 (4+3)	3	pT2c pN0 pMx	-	Yes
PDX#41	67	17	7 (4+3)	7 (4+3)	3	pT2c pN0 pMx	Yes (6)	
PDX#42	58	14	7 (4+3)	7 (3+4)	2	pT2c pNx pMx	-	
PDX#43	49	5	7 (3+4)	7 (3+4)	2	pT2c pNx pMx	-	Yes
PDX#44	73	11.1	7 (4+3)	7 (3+4)	2	pT3a pNx pMx	-	Yes
PDX#45	72	8.1	8 (4+4)	7 (4+3)	3	pT2c pNx pMx	Yes (18)	Yes
PDX#46	76	5.9	7 (3+4)	7 (3+4)	2	pT2c pNx pMx	-	Yes
PDX#47	61	8.2	6 (3+3)	6 (3+3)	1	pT2c pNx pMx	-	Yes
PDX#48	57	6.5	7 (3+4)	7 (3+4)	2	pT2c pNx pMx	-	Yes
PDX#49	75	6.2	8 (4+4)	7 (4+3)	3	pT2c pNx pMx	Yes (2)	Yes
PDX#50	79	7	7 (3+4)	7 (3+4)	2	pT3a pNx pMx	-	Yes
PDX#51	72	9.8	7 (4+3)	7 (3+4)	2	pT2c pNx pMx	-	Yes
PDX#52	70	6	6 (3+3)	6 (3+3)	1	pT2c pNx pMx	-	Yes
PDX#53	73	14.4	10 (5+5)	7 (4+3)	3	pT3b pNx pMx	Yes (5)	Yes
PDX#54	71	11.7	7 (4+3)	7 (3+4)	2	pT2c pNx pMx	-	Yes

PDX#55	78	6.8	7 (3+4)	7 (3+4)	2	pT2c pNx pMx	-	
PDX#56	66	12	6 (3+3)	7 (3+4)	2	pT2c pNx pMx	-	Yes
PDX#57	64	0.7	7 (3+4)	7 (3+4)	2	pT2c pNx pMx	-	
PDX#58	68	3.6	7 (3+4)	7 (3+4)	2	pT2c pNx pMx	Yes (5)	Yes
PDX#59	61	5.4	7 (4+3)	7 (4+3)	3	pT2c pN0 pMx	-	Yes
PDX#60	66	17	9 (4+5)	7 (4+3)	3	pT3b pN0 pMx	Yes (2)	Yes
PDX#61	69	29	6 (3+3)	6 (3+3)	1	pT2c pNx pMx	-	Yes
PDX#62	65	11	7 (3+4)	7 (3+4)	2	pT3a pNx pMx	-	Yes
PDX#63	65	8	7 (3+4)	7 (3+4)	2	pT2c pNx pMx	-	Yes
PDX#64	69	4.8	7 (3+4)	7 (4+3)	3	pT3b pNx pMx	Yes (3)	Yes

* Prostate cancer grade group according ISUP (International Society of Urological Pathology)
